# Supplementary material for: Photoinitiator Selection and Concentration in Photopolymer Formulations towards Large-Format Additive Manufacturing
Source: Polymers (Basel). 2022 Jul 1;14(13):2708. doi: 10.3390/polym14132708 (PMC9268840; doi:10.3390/polym14132708)
Supplement: Supplementary file 1 [file polymers-14-02708-s001.zip › polymers-1726243-supplementary.pdf]

Supplemental Information

BAPO Reaction

Bis(2,4,6-trimethylbenzoyl)-phenylphosphine oxide is a common photobleaching photoinitiator, also known as BAPO and sold under the trade name Omnirad 819. Upon exposure to UV light, the molecule undergoes a Type I scission reaction, separating into a phosphinoyl radical and a benzoyl radical (see Figure S1). Both radicals are highly reactive and can react with acrylate/methacrylate pendant groups to initiate a polymerization reaction.

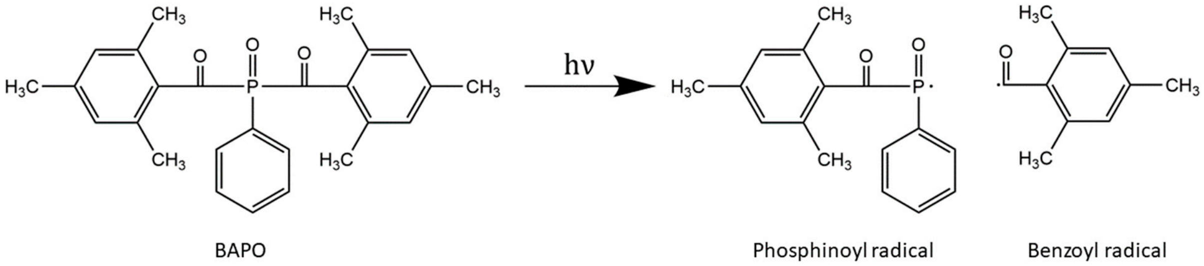

Figure S1 Reaction byproducts of BAPO upon exposure to UV light (adapted from [1], [2]).

UV-Vis Spectroscopy

The commercial-grade Bis-EMA (CG Bis-EMA) used throughout the study contains impurities such as methacrylic acid that are typically removed or reduced in a higher purity dental-grade Bis-EMA such as X-970-0000 (Esstech Inc., Essington, PA, USA) . To further investigate the potential impact of these impurities on light transmission, absorption properties were determined for both CG Bis-EMA and X-970-0000 (referred to as DG Bis-EMA) using an Evolution 300 spectrometer from Thermo Fisher Scientific (Waltham, MA, USA), with a scan range of 300 to 500 nm and a bandwidth of 2.0 nm. Resins were dissolved at 5 wt% in spectroscopy-grade acetone (Uvasol®, MilliporeSigma, Burlington, MA, USA) and solutions were tested in a 3500 microliter quartz cuvette with a lid from Thorlabs, Inc (Newton, NJ, USA). Due to the absorbance range of acetone, only the absorbance >325 nm could be used.

As shown in Figure S2, the CG Bis-EMA had the highest light absorbance of the resins tested, while the DG Bis-EMA had very low light absorbance. The addition of 20 wt% PETIA reduced the absorbance in the CG Bis-EMA:PETIA blend used throughout the study, and these values were used in the optical model as A_{mj} values.

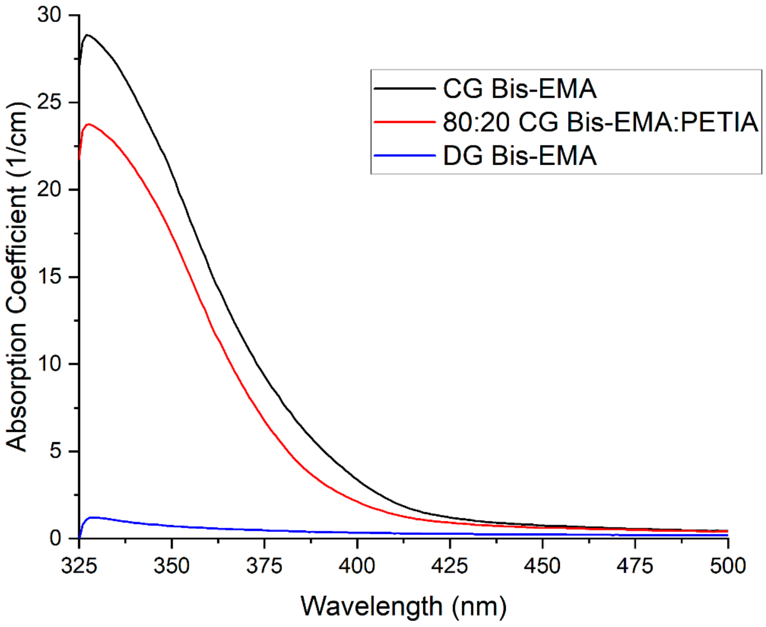

Figure S2 UV-Vis absorption spectrum for two grades of Bis-EMA and the 80:20 Bis-EMA:PETIA blend used throughout the study.

Radiometry

Light transmission through 3 mm neat resin was monitored with the Altair 75 UV LED system, under an incident light intensity of 380 mW/cm^2. As shown in Figure S3, DG Bis-EMA has the highest light transmission and maintains near constant transmission of 78% of the incident light intensity throughout 60 s irradiation time. The CG Bis-EMA used throughout the study has higher absorbance due to residual methacrylic acid and possible other unidentified impurities, which results in much lower transmission. With 20 wt% PETIA added, the transmitted light intensity improves above the baseline CG Bis-EMA but is only 29% at 60 s. Note that the transmission of the resins containing CG Bis-EMA increases over time with UV exposure, which is due to the curing reaction induced in CG Bis-EMA without the presence of any initiator. The hypothesis is that this reaction is due to the residual methacrylic acid content, which has

functional groups that can cause a polymerization reaction. This initiator-free polymerization was not observed in the DG Bis-EMA, which has a lower residual methacrylic acid content and inhibitors. The inhibitor package of the CG Bis-EMA is unknown, and it is unclear whether the initiator-free polymerization is due to the methacrylic acid absorbing light and reacting, or if other impurities are absorbing light and potentially inducing a thermal reaction.

The Radiometry results confirm the trends observed in the UV-Vis results, with residual methacrylic acid content and other contaminants leading to higher UV absorption. This is significant because it will limit overall light transmission through a thick polymer system. Due to a higher absorption coefficient, the CG Bis-EMA used in the present study has higher light attenuation than a DG Bis-EMA. However, CG Bis-EMA is apposite for LFAM applications, as it is readily available in large volumes and at a lower cost than DG Bis-EMA.

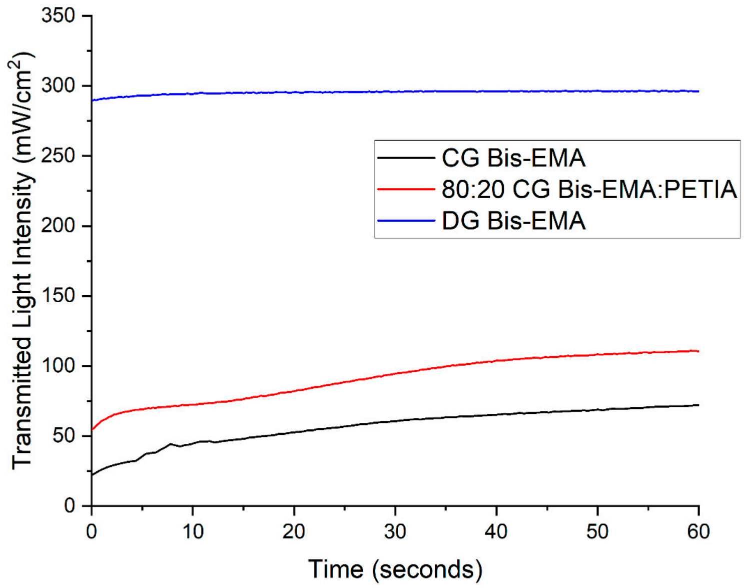

Figure S3 Transmitted light intensity for two grades of Bis-EMA and the 80:20 Bis-EMA:PETIA blend used throughout the study.

References

[1] W. A. Greene, *Industrial Photoinitiators*, 1st ed. Boca Raton: Taylor & Francis, 2010.

[2] K. Ruhland, F. Habibollahi, and R. Horny, "Quantification and elucidation of the UV-light triggered initiation kinetics of TPO and BAPO in liquid acrylate monomer," *J. Appl. Polym. Sci.*, vol. 137, no. 6, p. 48357, Feb. 2020.
